# An Implantable Inductive Near-Field Communication System with 64 Channels for Acquisition of Gastrointestinal Bioelectrical Activity

**DOI:** 10.3390/s19122810

**Published:** 2019-06-24

**Authors:** Amir Javan-Khoshkholgh, Aydin Farajidavar

**Affiliations:** Integrated Medical Systems (IMS) Laboratory at the College of Engineering and Computing Sciences, New York Institute of Technology, Old Westbury, NY 11568, USA; ajavankh@nyit.edu

**Keywords:** gastric slow waves, high-resolution mapping, near-field data communication, wireless power transfer, implantable medical devices

## Abstract

High-resolution (HR) mapping of the gastrointestinal (GI) bioelectrical activity is an emerging method to define the GI dysrhythmias such as gastroparesis and functional dyspepsia. Currently, there is no solution available to conduct HR mapping in long-term studies. We have developed an implantable 64-channel closed-loop near-field communication system for real-time monitoring of gastric electrical activity. The system is composed of an implantable unit (IU), a wearable unit (WU), and a stationary unit (SU) connected to a computer. Simultaneous data telemetry and power transfer between the IU and WU is carried out through a radio-frequency identification (RFID) link operating at 13.56 MHz. Data at the IU are encoded according to a self-clocking differential pulse position algorithm, and load shift keying modulated with only 6.25% duty cycle to be back scattered to the WU over the inductive path. The retrieved data at the WU are then either transmitted to the SU for real-time monitoring through an ISM-band RF transceiver or stored locally on a micro SD memory card. The measurement results demonstrated successful data communication at the rate of 125 kb/s when the distance between the IU and WU is less than 5 cm. The signals recorded in vitro at IU and received by SU were verified by a graphical user interface.

## 1. Introduction

Gastrointestinal (GI) peristalsis is coordinated by an underlying bioelectrical activity, known as slow waves (SWs). Alvarez conducted pioneering studies in acquiring the SWs in 1920s [1]. SWs either directly taken from the stomach or the abdominal skin have been proven to be an indicator of the peristalsis. Direct recording of the SWs demonstrated a robust acquisition, and acceptable signal to noise ratio compared to non-invasive recordings [2]. The information that can be extracted from single channel recording of SWs is limited; as a result, high-resolution (HR) mapping has been employed in recent years [3]. HR mapping of the SWs has been shown to be an effective tool for accurately defining functional motility disorders such as gastroparesis, chronic nausea, and functional dyspepsia [4,5,6,7]. Howsoever, current HR mapping methods are invasive, and the SWs are recorded directly from the serosa layer of a fasted subject undergoing abdominal surgery. Therefore, all the HR mapping studies have been based on short-term studies while the subject was under anesthesia. In addition to discomfort or risk of dislodgement or infection for the patient caused by current recording devices, a major limitation of these systems is their shortcoming for long-term studies.

Several studies have tried to employ wireless technologies to develop an implantable device suitable for HR mapping of the SWs. The first generations of such systems with limited number of channels (three to seven channels) were developed and validated by [8,9,10]. The number of channels in these systems were sufficient to map the activity of the stomach while functioning normally; however, when the stomach activity was ectopic, seven channels were not enough to capture the direction of the SWs propagation [11]. Using off-the-shelve components, we developed a 32-channel system to accommodate this shortcoming [12,13]. The power consumption of these systems was in the range 30 mW, making them unsuitable for long-term studies. Furthermore, none of these systems were equipped with a recharging mechanism, in case they need to be implanted.

With the objective of designing a minimally invasive system for HR mapping, we have developed, and benchtop validated, a 64-channel recording system that is composed of an implantable unit (IU), a wearable unit (WU), and a stationary unit (SU). The IU conditions the SWs, and the WU reads the SWs from the IU through a radio frequency identification (RFID) -based near-field communication (NFC) system [14,15], and consequently, transmits the data to the SU. Furthermore, the WU can wirelessly transfer power to the IU through an inductive link. The low power consumption of the IU, and the recharging feature allows implantation of the IU for long-term studies.

In the following, we first describe the NFC methodology consisting of data encoding algorithm and data modulation method. Afterwards, the system architecture and implementation of the SU, the WU, and the IU devices are explained. At the end, the validation of the IU-WU wireless data communication and wireless power transfer (WPT) and the WU-SU RF link, followed by a discussion of the results, is presented.

## 2. Near-Field Communication Methodology

**Figure 1:**
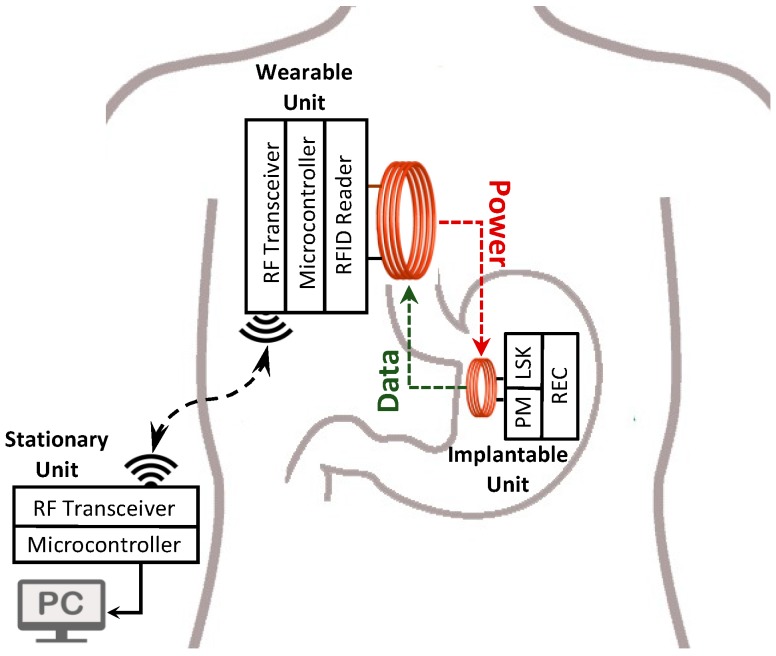
The block diagram of the near-field communication system for simultaneous wireless power transfer and telemetric acquisition of the gastric bioelectrical activity is shown.

The block diagram of the closed-loop wireless power and data transmission system is presented in Figure 1. The NFC between the IU and WU is implemented inductively through a 13.56 MHz RFID link. The WPT from the WU’s coil to the IU’s coil and wireless data telemetry on the reverse link are carried out through a single inductive coupling link at the carrier frequency, i.e., 13.56 MHz. Since the NFC and WPT are performed simultaneously over the same link, the WPT efficiency decreases significantly. As a result, data telemetry has to be executed in narrow pulses. The use of narrow pulses for data communication would minimize the disruption of the inductive link and maximize the efficiency of the WPT. In addition, the lack of two independent links for sending data packets and clock signal for the data telemetry is the second challenge in IU-WU NFC with only one inductive link. To solve the problem and to synchronize the data at IU and WU sides, a customized self-clocking data encoding algorithm was developed and validated. 

### 2.1. Wireless Data Transmission

#### 2.1.1. Differential Pulse Position (DPP) Data Encoding

Manchester encoding is a self-clocking data transmission method in which the data signal is changed in the middle of every bit [16]. According to IEEE 802.3 standard, logic “1” is encoded by the data signal transition from low to high in the middle of the bit and remains high; and for logic “0” the signal initially goes from high to low and remains low. However, Manchester encoding requires a duty cycle of 50%, which dramatically drops the WPT efficiency. Therefore, to minimize high pulses of the transmitting data, a differential pulse position (DPP) encoding method was proposed to guarantee very-low duty cycle of each data bit. Based on the developed algorithm, logic “1” is represented through the change of data signal from low to high in the middle of bit and is kept high for only 0.5 µs. Logic “0” is represented by only a 0.5 µs pulse at the beginning of the bit. 

The decision on the width of the high-pulses is made based on the minimum timing achievable by the microcontroller. Furthermore, the minimum time period of the encoded data bits at the transmitter side is limited by the envelope detector response time and the decoding accuracy of the data at the receiver side. Consequently, for the high-pulse and bit period times, 0.5 µs and 8 µs were chosen as optimum timings, respectively.

**Figure 2:**
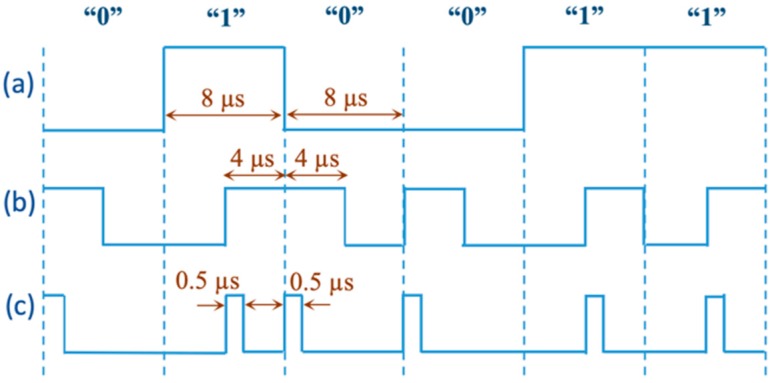
The schematic of the developed encoding algorithm is shown. (**a**) High and low digital logic values: “0” and “1”, (**b**) IEEE 802.3 standard Manchester encoding with 50% duty cycle, and (**c**) developed differential pulse position encoding with only 0.5 µs high-pulse width.

As a result, while the encoded data will be seen as successive pulses in varying time intervals; the duty cycle of the data signal is reduced from 50% to 6.25% (high-pulses are decreased from 4 µs to 0.5 µs) and the WPT continuity significantly increased from 50% to 93.75%. For instance, if the duration of each bit is assumed to be 8 µs with 50% duty cycle to present logic “1”, using the proposed method, we can present logic “1” with a narrow pulse of 0.5 µs reducing the duty cycle to 6.25%. The schematic of the data encoding is presented in Figure 2. Consequently, the encoder takes the data and generates the pulse stream according to the proposed method. Furthermore, a start-of-frame (SOF) is added as the packet header to identify the data stream at the decoder side. We used the sequence of “10000001” as the SOF. Upon receiving the data transmitted through the back-telemetry circuit at the IU, data decoding at the WU is proceeded based on time intervals between each two adjacent high pulses. The flowchart of the decoding algorithm was described in our previous work, in detail [17].

#### 2.1.2. Load-Shift Keying (LSK) Data Modulation

**Figure 3:**
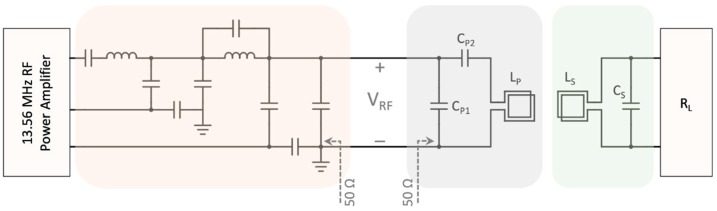
The circuit schematic for the analysis of load-shift keying data modulation is shown. L_P_ shows the primary coil matched to 50 Ω with C_P1_ and C_P2_, and L_S_ presents the secondary coil with the resonant capacitor of C_S_.

In an inductively coupled link consisting of two coils, changes of the impedance in one of the coils is reflected back in the other one. Therefore, since constant RF power is delivered to the transmitter coil with a 50 Ω matching network, any changes in the load of the secondary coil affects the voltage level of the primary coil. According to Figure 3, $Z_{secondary}$ is the impedance observed at the IU’s coil,

(1) $$\begin{array}{l} Z_{Secondary}=Z_{L_{S}}+(Z_{C_{S}}\;||\;R_{L})\\ Z_{Secondary}=\left(R_{S}+j\omega L_{S}\right)+\left(\frac{1}{j\omega C_{S}+\frac{1}{R_{L}}}\right)\\ Z_{secondary}=\left(R_{S}+\frac{R_{L}}{1+\omega^{2}\;C_{S}^{2}\;R_{L}^{2}}\right)+j\omega \left(L_{S}-\frac{R_{L}^{2}\;\;C_{S}}{1+\omega^{2}\;C_{S}^{2}\;R_{L}^{2}}\right) \end{array}$$

in which, $R_{S}$ is the internal resistance of the secondary coil. At the resonant frequency of *ω* = *ω*_0_ in Equation (1), the imaginary part of $Z_{Secondary}$ will be zero. So,

(2) $$L_{S}-\frac{R_{L}^{2}\;\;C_{S}}{1+\omega_{0}^{2}\;C_{S}^{2}\;R_{L}^{2}}=0$$

As a result,

(3) $$\frac{R_{L}}{1+\omega_{0}^{2}\;C_{S}^{2}\;R_{L}^{2}}=\frac{L_{S}}{R_{L}\;C_{S}}$$

Based on Equation (3), the impedance of the secondary side at the resonant frequency is,

(4) $$\begin{array}{l} Z_{Secondary\left(\omega_{0}\right)}=R_{S}+\frac{R_{L}}{1+\omega_{0}^{2}\;C_{S}^{2}\;R_{L}^{2}}\\ Z_{Secondary\left(\omega_{0}\right)}=R_{S}+\frac{L_{S}}{R_{L}\;C_{S}} \end{array}$$

**Figure 4:**
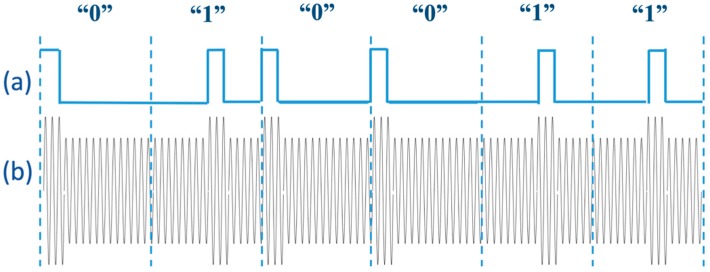
The schematic of the load-shift keying data modulation is shown. (**a**) The digital sequences of “0”s and “1”s are encoded by differential pulse position algorithm at the implantable unit, and (**b**) the encoded data modulated over 13.56 MHz carrier signal, can be seen by the envelope detector at the wearable unit. It only presents the concept and does not take into consideration instantaneous possible changes of the transmitting power.

The impedance of the secondary side reflected at the primary coil is,

(5) $$\begin{array}{l} Z_{reflected\left(\omega_{0}\right)}=\frac{\omega_{0}^{2}\;M^{2}}{Z_{Secondary\left(\omega_{0}\right)}}\\ Z_{reflected\left(\omega_{0}\right)}=\frac{\omega_{0}^{2}\;M^{2}}{\left(R_{S}+\frac{L_{S}}{R_{L}\;C_{S}}\right)} \end{array}$$

in which, M is the mutual inductance between the primary and secondary coils. The reflected impedance of the secondary side in Equation (5) is directly proportional to the load resistance connected to the secondary coil. In this regard, load-shift keying (LSK) modulation can be implemented by switching on and off a load (ground connection) at the IU’s coil through an NMOS transistor switch, which is reflected as the changes in the impedance of the WU’s coil and consequently, modifies the voltage level of the RF power signal. Since the primary coil is matched to 50 Ω, when the switch is open, the reflected impedance detunes the matching network and slightly decreases the voltage amplitude at the WU’s coil. Otherwise, for sending 0.5 µs high-pulses of the DPP encoded data, the switch is closed, zero resistance is reflected from the secondary coil and so, the voltage at the primary coil increases to the level corresponding to 50 Ω tuned matching network. At the WU, an envelope detector circuit detects the voltage level variations and demodulates the transmitted data, accordingly. The schematic of the data modulated over the RF carrier signal is shown in Figure 4.

### 2.2. Wireless Power Transfer

#### 2.2.1. The Transmitter Coil’s Matching Network

The RF power amplifier on the WU (embedded in the RFID reader chip) can deliver its nominal power to a 50 Ω load. It provides the possibility of using independent transmitter coils with various dimensions tuned at 50 Ω matching networks. Since the coils are of inductive load type, a capacitive tuning circuit for the matching network is offered to enhance the quality (Q) factor. The matching circuit consisting of a series tuning capacitor (C_P2_) and a parallel load capacitor (C_P1_) is presented in Figure 3. The impedance of the transmitter coil with the capacitive matching network is,

(6) $$\begin{array}{l} Z_{Primary}=\left(Z_{C_{P2}}+Z_{L_{P}}+R_{P}\right)\;||\;Z_{C_{P1}}\\ Z_{Primary}=\frac{1}{\left(\frac{1}{\frac{1}{j\omega C_{P2}}+j\omega L_{P}+R_{P}}\right)+j\omega C_{P1}}\\ Z_{Primary}=\frac{1}{\omega C_{P1}}\left(\frac{R_{P}{\left(\frac{1}{\omega C_{P1}}\right)}^{2}-j\;\left(R_{P}^{2}+\left(\omega L_{P}-\frac{1}{\omega C_{P2}}\right)\left(\omega L_{P}-\left(\frac{1}{\omega C_{P2}}+\frac{1}{\omega C_{P1}}\right)\right)\right)}{R_{P}^{2}+{\left(\omega L_{P}-\left(\frac{1}{\omega C_{P2}}+\frac{1}{\omega C_{P1}}\right)\right)}^{2}}\right) \end{array}$$

in which $R_{P}$ is the internal resistance of the primary coil. Since $Z_{Primary}$ has to be matched to 50 Ω, the imaginary part of the Equation (6) will be zero. Therefore,

(7) $$R_{P}^{2}+\left(\omega_{0}L_{P}-\frac{1}{\omega_{0}C_{P2}}\right)\left(\omega_{0}L_{P}-\left(\frac{1}{\omega_{0}C_{P2}}+\frac{1}{\omega_{0}C_{P1}}\right)\right)=0$$

As a result,

(8) $$Re\left(Z_{Primary}\right)=\frac{R_{P}{\left(\frac{1}{\omega_{0}C_{P1}}\right)}^{2}}{-\left(\frac{1}{\omega_{0}C_{P1}}\right)\left(\omega_{0}L_{P}-\left(\frac{1}{\omega_{0}C_{P2}}+\frac{1}{\omega_{0}C_{P1}}\right)\right)}$$

Besides, since the real part of $Z_{Primary}$ will be equal to 50 Ω,

(9) $$\omega_{0}L_{P}-\frac{1}{\omega_{0}C_{P2}}=\frac{1}{\omega_{0}C_{P1}}\left(1-\frac{R_{P}}{50}\right)$$

By substituting Equation (9) into Equation (7), we obtain

(10) $$C_{P1}=\frac{1}{50\;\omega_{0}\;\sqrt{\frac{R_{P}}{50-R_{P}}}}$$

Also, by substituting Equation (10) into Equation (9), we find

(11) $$C_{P2}=\frac{1}{\omega_{0}\left(\omega_{0}L_{P}-\left(50-R_{P}\right)\sqrt{\frac{R_{P}}{50-R_{P}}}\right)}$$

#### 2.2.2. The Receiver Coil’s Resonant Network

To maximize the receiving power transferred through the transmitter coil, the LC parallel network at the IU is to be tuned at the resonant frequency of 13.56 MHz. Taking into consideration the internal resistance of the receiver coil, the impedance of the secondary LC tank is,

(12) $$\begin{array}{l} Z_{L_{S}C_{S}}=Z_{L_{S}}||\;Z_{C_{S}}\\ Z_{L_{S}C_{S}}=\frac{1}{\left(\frac{1}{R_{S}+j\omega L_{S}}+j\omega C_{S}\right)}\\ Z_{L_{S}C_{S}}=\left(\frac{\frac{R_{S}L_{S}}{C_{S}}-\frac{R_{S}}{\omega C_{S}}\left(\omega L_{S}-\frac{1}{\omega C_{S}}\right)}{R_{S}^{2}+{\left(\omega L_{S}-\frac{1}{\omega C_{S}}\right)}^{2}}\right)-j\left(\frac{\frac{L_{S}}{C_{S}}\left(\omega L_{S}-\frac{1}{\omega C_{S}}\right)+\frac{R_{S}^{2}}{\omega C_{S}}}{R_{S}^{2}+{\left(\omega L_{S}-\frac{1}{\omega C_{S}}\right)}^{2}}\right) \end{array}$$

Since the imaginary part of the LC tank impedance at the resonance frequency is zero,

(13) $$\frac{L_{S}}{C_{S}}\left(\omega_{0}L_{S}-\frac{1}{\omega_{0}C_{S}}\right)+\frac{R_{S}^{2}}{\omega_{0}C_{S}}=0$$

As a result,

(14) $$\omega_{0}^{2}=\frac{1}{C_{S}\;L_{S}}-{\left(\frac{R_{S}}{\;L_{S}}\right)}^{2}$$

Therefore, by measuring the secondary coil’s inductance and resistance, the resonance capacitance can be obtained from Equation (14),

(15) $$C_{S}=\frac{\frac{1}{L_{S}}}{\;\omega_{0}^{2}+{\left(\frac{R_{S}}{\;L_{S}}\right)}^{2}}$$

## 3. System Architecture

**Figure 5:**
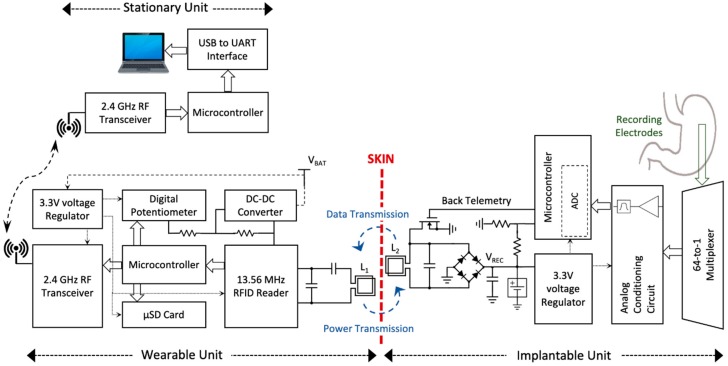
The detailed block diagram of the system consisting of the implantable, wearable and stationary units for near-field communication and wireless power transfer is presented.

The detailed block diagram of the NFC and WPT system is shown in Figure 5, which consists of the IU, the WU, and the SU connected to a computer. The WPT feature is carried out by inductively transmitting the 13.56 MHz sinewave signal generated by the power amplifier of an RFID reader from the WU’s coil to the IU’s coil (L_1_ to L_2_). This signal is rectified and regulated at 3.3 V on the IU. For the NFC, an ADC embedded in a microcontroller at the IU reads the GEA signals from 64 electrodes consecutively which are multiplexed through a 64-to-1 multiplexing circuit and processed through an analog conditioning circuit, and digitizes the data with a sampling rate of 16 samples per second per channel. Afterward, the microcontroller applies the proposed DPP encoding algorithm to the data packet and drives the NMOS switch with the data pulse train of every 64-channel signals. At the WU, the data are demodulated by an envelope detector embedded in the RFID reader (TRF7970, Texas Instruments -TI-) and decodes them by the microcontroller. Although the system architecture was briefly described in our previous work [18], the specific details are discussed here.

### 3.1. IU Data Packet

In addition to the digitized data of 64 signal acquisition channels, the instantaneous rectified voltage of received power by the IU (V_REC_) and the IU’s battery voltage are concatenated in one data packet. As a result, based on the ADC sampling resolution of 10 bits, the data packet includes 640 bits for samples of 64 signal acquisition channels, 10 bits for the rectified voltage and 10 bits for the battery voltage. Furthermore, a start-of-frame of 8 bits is added as the header of the packet to make the decoder circuit capable of identifying the data stream sent to the WU. As a result, the final data packet consists of 668 bits per each round of acquiring 64 GEA signals. Speaking in terms of the time required to transmit the data through LSK modulation, the back-telemetry circuit is off for around 64 ms (1 ms is the multiplexing circuit delay time to let the ADC to acquire the input signal of 64 independent channels) and then takes 668 × 8 µs equal to 5.344 ms to send the NFC data. However, since the duty cycle of the DPP encoded data is 6.25%, the wireless power receiving at the IU is only off for around 0.334 ms during the NFC. Table 1 presents the content of a data packet transmitted from the IU to the WU. 

### 3.2. Closed-Loop WPT

Stomach motility or body movements of the subject undergoing the study can potentially result into misalignment and/or changes of distance between the primary and secondary coils. These, in turn, can significantly affect the amount of power received by the IU and decrease the efficiency of WPT. As a result, a closed-loop system for the WPT is designed to guarantee the minimum amount of power receiving by the IU and consequently, to adjust the transmitting power by the WU, accordingly. To implement the closed-loop WPT, instantaneous V_REC_ at the IU is read through a voltage divider and sampled by an ADC of the IU’s microcontroller (MSP430, TI) and as indicated in Table 1, is sent to the WU as a part of the GEA data packet. At the WU, the DC bias voltage of the RF power amplifier is supplied through the output of a DC-DC buck-boost converter (TPS63000, TI). The feedback resistor of the buck-boost converter is a 200 kΩ digital potentiometer with 256 taps (MAX5424, Maxim Integrated) which is programmable through the WU’s microcontroller (MSP432, TI). At approximately every 70 ms, the WU receives the value of the instantaneous rectified voltage, the microcontroller compares it with a reference voltage (V_REF_) equal to 3.7 V. For V_REC_ voltages higher than V_REF_, the microcontroller increases the value of the digital potentiometer and consequently, decreases the output of the DC-DC buck-boost converter. As a result, the output power level of the RF power amplifier decreases until V_REC_ is decreased to V_REF_. On the other hand, when V_REC_ is lower than V_REF_, the digital potentiometer value decreases until the rectified voltage increases to V_REF_. The resistive feedback loop of the DC-DC buck-boost converter is designed such that it gives an output voltage range from 2.75 V to 5 V. 

The rectification bridge is a critical component in energy harvesting in wirelessly powered systems. Therefore, a Schottky diode array (BAS4002, Infineon, Neubiberg, Germany) employed at the IU is a low power and low forward voltage bridge with only 0.4 V voltage drop for 10–20 mA. As a result, the voltage decreases, in the worst case, 0.8 V in each direction.

### 3.3. Data Logging Modes

Once the GEA digitized signals are demodulated, and decoded at the WU, then the data can be either stored locally on a micro SD memory card or wirelessly transmitted to the SU through an ISM-band 2.4 GHz RF transceiver (nRF24L01+, Nordic Semiconductor). In the case of the former data logging mode (on the memory card), while the SU is out of the loop, the decoded data is saved in a text file on the memory card and can be extracted anytime. For the latter mode, data are received by the SU through the same RF transceiver and through a UART-to-USB bridge (FT232, FTDI Chip), are sent to the stationary computer. 

### 3.4. Graphical User Interface (GUI)

An application-specific GUI is developed in LabVIEW, it allows the user to monitor the 64 channels GEA signals in real time and store them on computer for off-line analysis. Hence, 64 independent signal windows with modifiable time intervals are embedded in the GUI. Instantaneous rectified and battery voltages are also displayed in the GUI. Furthermore, the user can either continue receiving the data through the RF link or switch to micro SD memory card data logging mode, wirelessly. A snapshot of the developed GUI is shown in the results section. 

## 4. Tests and Measurements Results

**Figure 6:**
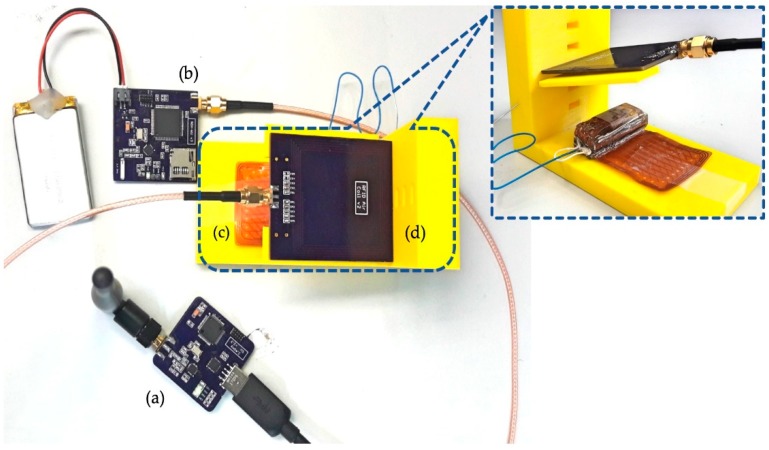
The implemented system for the validation of the 64-channel near-field communication signal acquisition and wireless power transfer, consisting of (**a**) the stationary unit connected to computer, (**b**) the wearable unit connected to a LiPo battery, (**c**) the implantable unit and (**d**) the transmitter coil. The inset shows the side view of the holder used to adjust various distances and angles between the primary and secondary coils.

To validate the high-resolution NFC signal acquisition and WPT systems, the setup shown in Figure 6 was implemented. The setup consists of the IU, the WU, the SU and a 3-D yellow-colored apparatus for the modification of the distances and the angles between the transmitter and receiver coils. The IU was fabricated on a 4-layer flex-rigid printed circuit board (PCB) with integrated flexible planar coil and integrated 64-channel flexible biocompatible electrode array. The IU (except the electrode array) was coated by a medical-grade epoxy (Epotek MED-301). The final size of the IU after coating was 13 × 11 × 40 mm^3^. Besides, the WU (45 × 45 mm^2^) and the SU (40 × 40 mm^2^), and the WU’s planar coil (53 × 62 mm^2^) were fabricated on regular 4-layer and 2-layer PCBs, respectively. 

### 4.1. Measurements Results of WPT

**Figure 7:**
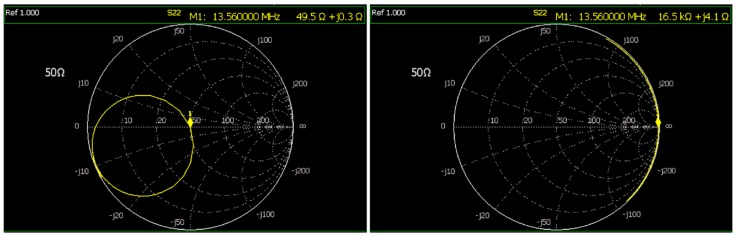
The impedances of (left) the wearable unit’s transmitter coil with the 50 Ω capacitive matching network equal to (49.5 + j0.3) Ω, (right) the impedance of the implantable unit’s receiver coil resonant LC network equal to (16.5 + j0.0041) kΩ, both measured at the RFID carrier frequency of 13.56 MHz.

Based on the analyses presented in Section 2.2.1 and Section 2.2.2, the 50 Ω capacitive matching network of the WU’s transmitter coil and the resonant network of the IU’s receiver coil were calculated, implemented and tuned at the RFID carrier frequency, i.e., 13.56 MHz. In this regard, we measured the inductances and internal resistances of the transmitter and receiver coils by a N9923A Vector Network Analyzer (VNA), independently. The inductance and outer diameter of the primary and secondary coils were measured as 3.02 µH and 490 nH, and 70 mm and 27 mm, respectively. Figure 7 shows the impedances of the transmitter coil matched to 50 Ω and the resonant LC tank at the receiver coil measured by the VNA. In particular, the L_1_ matched impedance of (49.5 + j0.3) Ω, provides an excellent return loss of around −30 dB, which guarantees the total amount of power generated by the RFID reader is delivered to the WU’s coil. 

**Figure 8:**
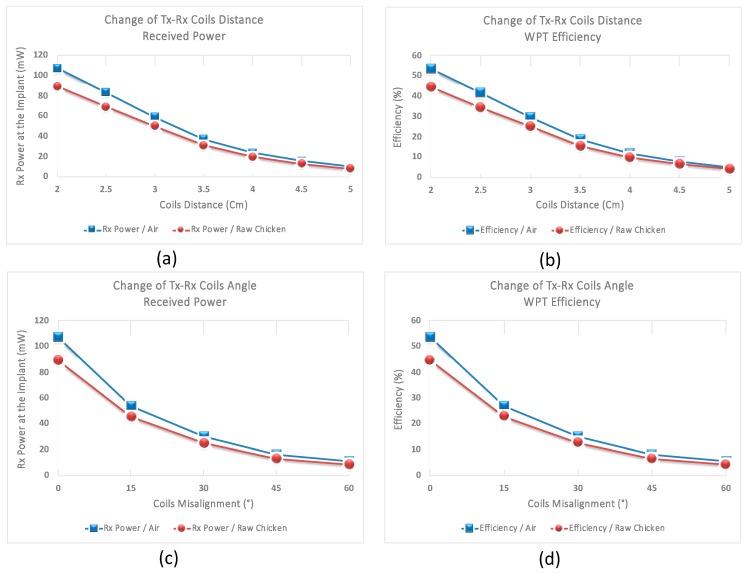
(**a**) and (**b**) show the received power and efficiency when the distance between the TX and RX coils is changed from 2 cm to 5 cm for air and raw chicken. (**c**) and (**d**) show the received power and efficiency when the alignment between the TX and RX coils is changed from 0° to 60° for air and raw chicken.

Furthermore, in order to verify the WPT, the WU was programmed to transmit constant power of 200 mW (23 dBm), and at the IU, the received power was measured for different distances and angles made between the pair of coils. For the medium between the primary and secondary coils, air and raw chicken were tested separately. Figure 8 presents the amount of the power received at the IU along with the WPT efficiency when the distance and the angle between the transmitter and the receiver coils were modified from 2 cm to 5 cm with the steps of 0.5 cm and from 0° to 60° with the steps of 15°, respectively. As shown in this figure, when the distance between the transmitter and receiver coils is 2 cm, the received power is 107 mW and 89 mW, for air and raw chicken, which translates to 53.5% and 44.5% efficiency, respectively. Increasing the distance from 2 cm to 5 cm, with the steps of 0.5 cm, the received power and the WPT efficiency eventually decrease to 10 mW and 8 mW, and to 5% and 4% for the corresponding mediums, respectively. Furthermore, at a constant distance of 2 cm between the pair of coils, when the misalignment angle between the primary and secondary coils is 15°, the received power for the air and raw chicken is 54 mW and 46 mW, which translates to 27% and 23% efficiency, respectively. Increasing the angle from 15° to 60°, with the steps of 15°, the received power and the WPT efficiency ultimately decrease to 10.5 mW and 9 mW, and to 5.6% and 4.1% for the corresponding mediums, respectively. 

Temperature increase and heat generation in human tissue is a major concern in WPT. While the maximum 200 mW power transmission is an order of magnitude lower than the allowed specific absorption rate (SAR) of the tissue which is 1.6 W/kg, we carried out three experiments to measure the power transfer and the heat generated in the two sides of the raw chicken at different alignments and distances. The first measurement was conducted for 36 h on 85 g of raw chicken, approximately 117 cm^3^, for the Tx-Rx aligned coils distance of 3.5 cm. The temperature on the outside edge of the chicken was 77.5 °F, and the ambient temperature was 76.5 °F. The second measurement was carried out for 14 h on 49.3 g of raw chicken, approximately 66 cm^3^, for the Tx-Rx aligned coils distance of 3.5 cm. The temperature on the outside edge of the raw chicken was 75.5 °F, and the ambient temperature was 75 °F. Finally, the third measurement was conducted for 4 h on 64 g of raw chicken, approximately 82 cm^3^, for the Tx-Rx misaligned coils distance of 3.5 cm and 45° misalignment angle. The temperature on the outside edge of the chicken was 78 °F, and the ambient temperature was 77.5 °F. 

### 4.2. Measurement Results of NFC Slow Waves Recording 

**Figure 9:**
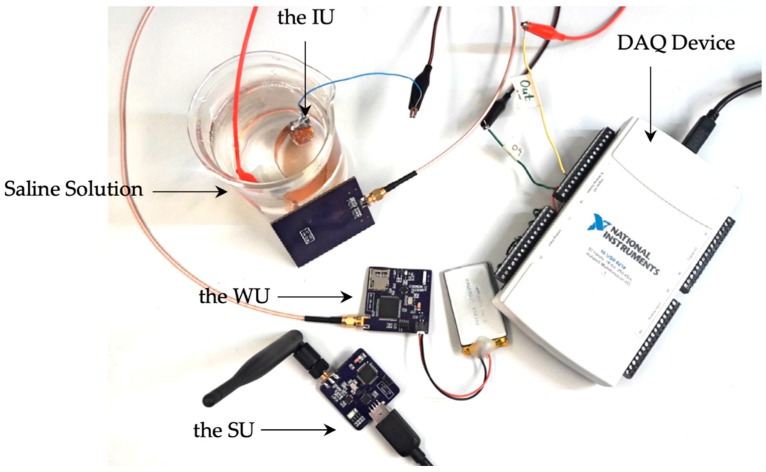
The benchtop setup for the verification of the near field communication recording is shown. A 5-min sample of slow waves recorded in vivo was loaded into a multifunction data acquisition device (DAQ USB-6218, National Instrument) and streamed into the implantable unit through saline solution. The signals were then recorded, sent to the wearable unit through the inductive link and wirelessly transmitted to the stationary unit.

For validation of the NFC recording system, the IU was drawn in a container of saline solution. In this test, 5-min sample SWs recorded previously *in-vivo* by our group were used [19]. Sample signals were loaded into a digital to analog converter (DAQ device USB-6218, National Instrument) through a custom-made application and were streamed into the solution. The data were verified in two steps. First, the consistency between the DPP-encoded data at the IU and the demodulated data which were recovered at the WU were verified. Then, the data received at the SU and monitored in the GUI in real time were observed to be identical with the amplified original signals recorded at the IU. The benchtop setup for the NFC verification is shown in Figure 9. 

**Figure 10:**
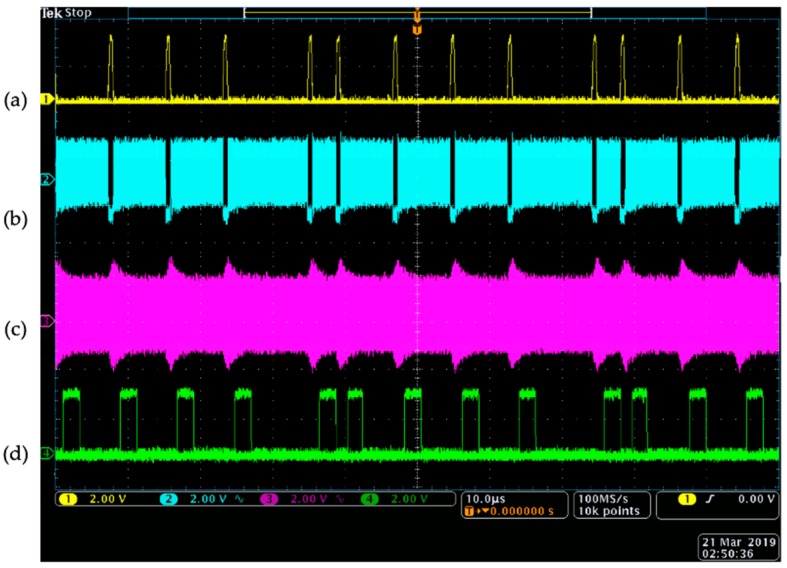
(**a**) The data encoded according to differential pulse position algorithm at the implantable unit, (**b**) the voltage of the secondary coil which drops to zero when the back-telemetry circuit sends a high-pulse of data, (**c**) the voltage of the primary coil which slightly increases when there is a change of impedance at the secondary coil, and (**d**) the demodulated data at the output of the RFID reader’s envelope detector. The time per division on the x-axis and the voltage per division on the y-axis for all four signals are 10 µs and 2 V, respectively.

At the WU, the received modulated data over the RF inductive link and the demodulated data at the output of the RFID reader were probed (using an oscilloscope, Tektronix MDO4104C) and simultaneously compared with the encoded data at the IU and the modulated data at the secondary coil. As presented in Figure 10, the power level at the IU’s coil drops to zero when the high-pulses of the encoded data are transmitted through the back-telemetry circuit. This is because the secondary coil is shorted to ground when the NMOS switch is closed. On the other side, when the modulated data is seen by the primary coil, the power level slightly increases. This is due to the fact that the reflected impedance to the WU’s coil is zero and as discussed in Section 2.1.2. in detail, the power level at the transmitter coil reaches its maximum possible value. Furthermore, it can be seen that the demodulated data at the output of the RFID reader successfully follows the exact time intervals in DPP-encoded data at the IU. 

**Figure 11:**
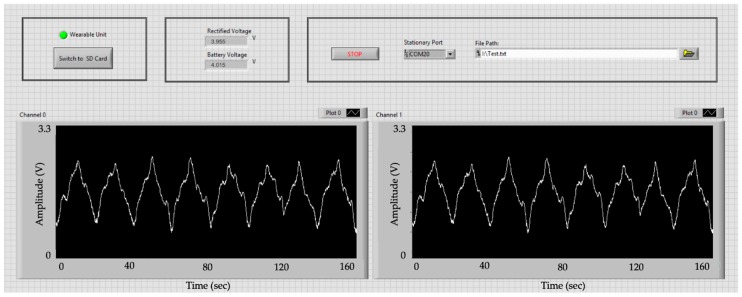
Signals received in the GUI is shown. *X* and *Y* axes are time (0 to 160 s) and amplitude (0 V to 3.3 V), respectively. Only two of 64 channels are shown, here. Eight slow-wave peaks in a time window of 160 s translates to 3 cycles per minute.

In the second test, the GEA data which were received in the 64 channels of the GUI in real time were monitored. Figure 11 shows the first two channels of the GUI received signals which are identical with the original ones; except that the received signal is amplified with the gain of 2000. Signals in other 62 channels of the GUI followed an identical pattern. Eight slow wave events can be counted in the period of 160 s which translates to 3 cycles per minute. 

The power consumption of the IU, WU, and SU were measured as 6.3 mW, 823 mW (at maximum power), and 80 mW, respectively. When the IU and WU coils are well-aligned and separated at 3 cm, the received power is enough to allow the IU to run without the need for a battery. However, because obtaining the perfect alignment of the coils in vivo is not easily achieved, the use of a battery for the IU seems necessary. Considering a 105 mAh Li-polymer battery (30 × 12 × 4 mm^3^) for the IU, the system can theoretically work for more than 15 continuous hours without the need for recharging. However, with the WPT charging capability when the coils were separated for 2.5 cm from each other (with perfect alignment), while the IU was in the container of saline solution and the WU’s coil was outside, the battery was recharged for 60 mV per hour, while working at maximum load.

## 5. Discussion and Conclusions

A wireless miniature implantable NFC system for high-resolution acquisition of SWs has been developed. The system is featured with a 13.56 MHz inductive RFID-based closed-loop NFC between the IU and the WU. The NFC works based on LSK modulation of data through a back-telemetry circuit. In addition, the system is equipped with WPT which assures a stable and solid source of power for the IU, and as a result, makes it an excellent candidate for long-term studies. Since the NFC and WPT are carried out simultaneously on one shared inductive link, an application-specific self-clocking DPP data encoding algorithm was developed. This algorithm utilizes very narrow width of the high-pulses with only 0.5 µs pulse width and duty cycle of 6.25% for encoding data to minimize the disruption of the WPT link. Furthermore, the proposed DPP encoding method provides the data rate of 125 kb/s, which is enough for our application. 

Two independent data logging modes were implemented in the system. The first mode is based on a 2.4 GHz RF link between the WU and the SU. Hence, the demodulated decoded data at the WU are transmitted to the SU connected to a computer. Through a GUI, the data can be monitored in real time and simultaneously stored in a text file for offline analysis. This method is preferred when the patient is under direct monitoring in a clinical facility. The second mode of data logging, which is wirelessly configurable through the GUI, is to store the data locally on the WU on a micro SD memory card. The data can be extracted from the memory card, anytime during or at the end of the GEA monitoring period.

The closed-loop feature of the system significantly improves the stability of the WPT. In case of any misalignment and change of distance between the primary and secondary coils due to body movement and stomach motility of the patient, the power transfer is instantaneously adjusted to keep the IU’s rectified voltage at a constant level. In addition, the range of 2 to 5 cm distance between the pair of coils is very reasonable for most of the subjects undergoing GI study, and the closed-loop WPT is capable of transmitting enough power to meet rectified voltage equal to reference voltage. The closed-loop WPT was intentionally limited to 200 mW that can be generated by the RFID reader. This amount is an order of magnitude lower than the specific absorption rate value allowed by the IEEE standard C95.1-2005 and International Commission on Non-Ionizing Radiation Protection (ICNIRP) guideline [20,21]. Furthermore, unnecessary higher amounts of power can generate noise that can reduces the fidelity of the signal transmission in the NFC link. 

The developed system with 64 signal acquisition channels covers an approximate area of 30 × 30 mm^2^. However, one might be interested to simultaneously study different areas of the gastrointestinal tract. In this regard, up to four systems can be employed simultaneously by modifying the carrier frequency of the RFID link. The default 13.56 MHz oscillator frequency of the RFID reader can be divided by 2 and 4, which means we can modify the clock frequency to 6.78 MHz and 3.39 MHz through the firmware. Moreover, we can replace the 13.56 MHz crystal oscillator of the RFID reader at the WU with the 27.12 MHz one. As a result, with minor modifications of the firmware of the IU and WU, we can concurrently run four systems with modulation frequencies of 3.39 MHz, 6.78 MHz, 13.56 MHz and 27.12 MHz.

For the closed-loop WPT, we considered the initial power transfer of 150 mW to the IU. The rectified voltage of the IU was measured continuously, and the algorithm in the WU was set to increase (or decrease) the transmitted power with 10 mW steps, in case the rectified voltage decreases (or increases) below (or above) the 3.7 V. This implementation is simple and does not consider various battery charging methods to enhance the performance of the battery. For instance, we can consider methods such as “constant current, constant voltage charging” or “multi-state current charging” algorithms, each of which have their advantageous and disadvantageous [22]. Using the closed-loop mechanism, more complicated algorithms for transmitting power to the IU can be developed in the future. 

Furthermore, the heating concern as a consequence of WPT through human tissue was studied in different distances and misalignment angles of the Tx-Rx coils. The measurements with maximum power transmission of 200 mW from the WU to the IU through raw chicken showed worst case temperature rise of 1 °F referenced to ambient, which is insignificant. In addition, regarding the blood circulation in a human’s stomach muscle, we are expecting to observe less temperature increase in practical study.

The power consumption of the NFC-based IU was measured as 6.3 mW that is far smaller than the counterpart 32-channel device (measured 48 mW), which utilizes a RF transceiver for data communication [12]. To even improve the power consumption further, one can consider developing the IU using application specific integrated circuit technology. Examples of such work can be found in [23,24]; however, this system is still in simulation phase. 

The IU’s dimension is 13 × 11 × 40 mm^3^. The IU has been designed to have a narrow width. The main reason is that we aspire to implant the device through a minimally-invasive procedure. We recently developed an endoscopic procedure for implantation of the device [25]. In this procedure, an endoscopic overtube is placed through the esophagus of the subject and the IU is then pushed inside the stomach through the tube to be implanted. Therefore, the diameter of the overtube, which corresponds to the diameter of the esophagus, is the determining factor of the width and thickness of the IU. The implant was designed such that the flexible electrode to be folded around the coated IU and the total diameter of the cross-section of the cylinder-shaped IU not to be greater than 18 mm.

Finally, In vitro studies demonstrated that the system can successfully record the signals akin to gastric SWs from 64 independent channels with a sampling rate of 16 samples per second per channel through the inductive NFC and recharge the IU’s local battery, simultaneously. We will conduct in vivo validation in pigs in the future, as their digestive system is similar to human.

## Figures and Tables

**Table 1:** Structure of the data packet to be encoded at the implantable unit.

	**Structure of a Data Packet**			
	**Header (SOF)**	**Data ^1^**		
	10000001	64 samples of acquired 64 signals	Rectified voltage sample	Battery voltage sample
	8 bits	640 bits	10 bits	10 bits
Packet data length	668 bits			
Packet time length	5.344 ms			
NFC Data Rate	125 kb/s			

^1^ The digitization resolution of the data at the implantable unit is 10 bits.
